# Supplementary material for: Predicting the spatio-temporal spread of West Nile virus in Europe
Source: PLoS Negl Trop Dis. 2021 Jan 7;15(1):e0009022. doi: 10.1371/journal.pntd.0009022 (PMC7790247; doi:10.1371/journal.pntd.0009022)
Supplement: S1 Table — (DOCX) [file pntd.0009022.s001.docx]

**S1 Table. Explanatory variables used in the WNV 2017 cases models in Europe.**

| **Type** | **Subtype** | **Abbreviation** | **Name** |
| --- | --- | --- | --- |
| Human | Human concentration | dens_pob | Population density^[1]^ |
|  |  | distcenpob | Distance to population center^[2]^ |
|  | Livestock | fao_chicken | Poultry density^[3]^ |
|  |  | fao_duck | Farmed duck density^[3]^ |
|  |  | fao_horse | Horse density^[3]^ |
|  |  | fao_pig | Pig density^[3]^ |
|  | Infrastructure | dist_road | Distance to roads^[4]^ |
|  |  | cor_urban | Continuous urban fabric^[5]^ |
|  |  |  | Discontinuous urban fabric^[5]^ |
|  |  |  | Industrial or commercial units^[5]^ |
|  |  |  | Construction sites^[5]^ |
|  |  | cor_road_rail | Road and rail networks and associated land^[5]^ |
|  |  | cor_grUrban | Green urban areas^[5]^ |
|  | Agriculture | cor_dry | Non-irrigated arable land^[5]^ |
|  |  |  | Vineyards^[5]^ |
|  |  |  | Olive groves^[5]^ |
|  |  |  | Annual crops associated with permanent crops^[5]^ |
|  |  | cor_rice | Rice fields^[5]^ |
|  |  | cor_irrig | Permanently irrigated land^[5]^ |
|  |  |  | Fruit trees and berry plantations^[5]^ |
|  |  |  | Pastures^[5]^ |
|  |  | cor_het_cult | Complex cultivation patterns^[5]^ |
|  |  | cor_agr_veg | Land principally occupied by agriculture with significant areas of natural vegetation^[5]^ |
|  |  | cor_agrfores | Agro-forestry areas^[5]^ |
| Non-human | Ecosystem | cor_broadleaf | Broad-leaved forest^[5]^ |
|  |  | cor_conif | Coniferous forest^[5]^ |
|  |  | cor_mixforest | Mixed forest^[5]^ |
|  |  | cor_grass | Natural grasslands^[5]^ |
|  |  | cor_moor | Moors and heathland^[5]^ |
|  |  | cor_scler | Sclerophyllous vegetation^[5]^ |
|  |  | cor_trans | Transitional woodland-shrub^[5]^ |
|  |  | cor_spars_veg | Sparsely vegetated areas^[5]^ |
|  |  | cor_in_marsh | Inland marshes^[5]^ |
|  |  | cor_peat | Peat bogs^[5]^ |
|  | Hydrographic | cor_sal_marsh | Salt marshes^[5]^ |
|  |  | cor_saline | Salines^[5]^ |
|  |  | cor_tidalFlat | Intertidal flats^[5]^ |
|  |  | cor_river | Water courses^[5]^ |
|  |  | cor_wat_body | Water bodies^[5]^ |
|  |  | cor_coast_lag | Coastal lagoons^[5]^ |
|  |  | cor_estuar | Estuaries^[5]^ |
|  |  | dist_rio | Distance to rivers^[6]^ |
|  | Topographic | alt | Altitude^[7]^ |
|  |  | slope | Slope^[8]^ |
|  | Climatic | Bio1 | Annual Mean Temperature^[9]^ |
|  |  | Bio5 | Max Temperature of Warmest Month^[9]^ |
|  |  | Bio6 | Min Temperature of Coldest Month^[9]^ |
|  |  | Bio7 | Temperature Annual Range (Bio5-Bio6) ^[9]^ |
|  |  | Bio12 | Annual Precipitation^[9]^ |
|  |  | Bio15 | Precipitation Seasonality (Coefficient of Variation)^9]^ |

^1^LandScanTM 2008 High Resolution Global Population Data Set (copyrighted by UT-Battelle, LLC, operator of Oak Ridge National Laboratory), excluding any areas less than 2-km far from urban areas (as delimited by the MODIS 500 -m Map of Global Urban Extent for 2001-2002 (Schneider et al. 2009; 2010).

^2^Administrative Centres & Populated Places shapefile at the Relational World Database II (RWDB2) updated in 2000 (http://www.fao.org/geonetwork).

^3^Global FAO 2010 livestock (http://www.fao.org/livestock-systems/en/).

^4^Vector Map Level 0 at the Digital Chart of the World (DCW, http://worldmap.harvard.edu), updated in 2002.

^5^Corine Land Cover 2018 (https://land.copernicus.eu/pan-european/corine-land-cover/clc2018).

^6^Global Drainage Basin Database GDBD. Released Version 1.0: May 29, 2007 ([http://www.cger.nies.go.jp/db/gdbd/gdbd_index_e.html](http://www.cger.nies.go.jp/db/gdbd/gdbd_index_e.html)).

^7^GTOPO30 (US Geological Survey 1996).

^8^Elaborated from DEM (Digital Elevation Model) using the altitude variable (GTOPO30; US Geological Survey 1996), using the Geographic Information Sistem ArcGIS Desktop 10.3.

^9^Chelsa (<http://chelsa-climate.org>).
