# Supplementary material for: Predicting the spatio-temporal spread of West Nile virus in Europe
Source: PLoS Negl Trop Dis. 2021 Jan 7;15(1):e0009022. doi: 10.1371/journal.pntd.0009022 (PMC7790247; doi:10.1371/journal.pntd.0009022)
Supplement: S2 Table — (DOCX) [file pntd.0009022.s002.docx]

**S2 Table**. **Explanatory variables included in the model of environmental favorability for the occurrence of WNF, based on cases of 2017**. The Wald parameter quantifies the relevance of every variable in the model. Variable abbreviations are given in S1 Table. B: Coefficients multiplying the variable values in the logit of the multivariate logistic regression. Sig.: Significance of the Wald test.

| **Variables** | **B** | **Wald** | **Sig.** |
| --- | --- | --- | --- |
| ***alt*** | -0.00187 | 5.92891 | 0.01489 |
| ***bio5*** | 0.01835 | 10.24530 | 0.00137 |
| ***bio7*** | 0.02008 | 13.22906 | 0.00028 |
| ***cor_dry*** | 2.10059 | 6.35829 | 0.01168 |
| ***cor_river*** | 42.97705 | 5.10371 | 0.02387 |
| ***fao_chicken*** | 0.0000070 | 5.42307 | 0.01987 |
| ***fao_horse*** | 0.00549 | 8.90469 | 0.00284 |
| ***Constant*** | -13.89171 | 89.67474 | 2.81E-21 |
