# Supplementary material for: Predicting the spatio-temporal spread of West Nile virus in Europe
Source: PLoS Negl Trop Dis. 2021 Jan 7;15(1):e0009022. doi: 10.1371/journal.pntd.0009022 (PMC7790247; doi:10.1371/journal.pntd.0009022)
Supplement: S3 Table — (DOCX) [file pntd.0009022.s003.docx]

**S3 Table.** **Combination of geographical coordinates included in the logit of the spatial favorability model for the occurrence of WNF, based on cases of 2017**. The Wald parameter quantifies the relevance of every variable in the model. La and Lo are latitude and longitude, respectively. B: Coefficients multiplying the variable values in the logit of the multivariate logistic regression. Sig.: Significance of the Wald test.

| **Variables** | **B** | **Wald** | **Sig.** |
| --- | --- | --- | --- |
| ***La*** | -94,999 | 20,926 | 0,0000048 |
| ***Lo*** | 9,366 | 6,223 | 0,013 |
| ***La^2^*** | 2,368 | 24,192 | 0,00000087 |
| ***La x Lo*** | -0,450 | 6,431 | 0,011 |
| ***La^2^ x Lo*** | 0,0054 | 6,732 | 0,0095 |
| ***La^3^*** | -0,020 | 27,216 | 0,00000018 |
| ***Constant*** | 1253,668 | 17,483 | 0,000029 |
