# Supplementary material for: Predicting the spatio-temporal spread of West Nile virus in Europe
Source: PLoS Negl Trop Dis. 2021 Jan 7;15(1):e0009022. doi: 10.1371/journal.pntd.0009022 (PMC7790247; doi:10.1371/journal.pntd.0009022)
Supplement: S4 Table — (DOCX) [file pntd.0009022.s004.docx]

**S4 Table. Explanatory variables included in the spatio-environmental favorability model for the occurrence of WNF, based on cases of 2017**. Ysp is the logit of a spatial logistic regression. The Wald parameter quantifies the relevance of every variable to the model. Variable abbreviations are given in S1 Table. B: Coefficients multiplying the variable values in the logit of the multivariate logistic regression. Sig.: Significance of the Wald test.

| **Variables** | **B** | **Wald** | **Sig.** |
| --- | --- | --- | --- |
| ***bio5*** | 0.01861 | 9.14733 | 0.00249 |
| ***cor_dry*** | 2.23346 | 8.64224 | 0.00328 |
| ***cor_river*** | 63.18861 | 4.69134 | 0.03031 |
| ***fao_chicken*** | 0.0000099 | 7.23869 | 0.00713 |
| ***Ysp*** | 0.85301 | 34.39060 | 4.51E-09 |
| ***Constant*** | -6.58993 | 16.20105 | 0.000057 |
