# Supplementary material for: Predicting the spatio-temporal spread of West Nile virus in Europe
Source: PLoS Negl Trop Dis. 2021 Jan 7;15(1):e0009022. doi: 10.1371/journal.pntd.0009022 (PMC7790247; doi:10.1371/journal.pntd.0009022)
Supplement: Attachment — Submitted filename: Authors response.docx [file pntd.0009022.s005.docx]

**Authors’ response**

Dear Editors:

We are submitting our revised manuscript, in which we added additional explanations in response to the insightful comments and advices given by the reviewers. We highly appreciate their contributions because taken them into account we have improved the manuscript, both the scope and readability.

Below you will find the comments, followed by our response in italicized and bold type.

With kind regards, also on behalf of the authors,

Reviewer #1:

The objectives of the study are clear, the study design is appropriate to address the stated objectives. Analyses used in the study were correct to support the conclusions.

- ***We appreciate the comments of the reviewer.***

The results are clearly and completely presented.

- ***We are very grateful for this comment.***

Conclusions supported the presented data. Authors also discuss how these data can be helpful to predict the locations of WNF outbreaks better (e.g.spatio-environmental model) and the environmental model predicts the intensity of the WNV cases.

- ***We appreciate the comments of the reviewer.***

The influence of precipitation in the spring and early summer month would also be interesting to consider. The considered variables in the development of the models are annual precipitation and precipitation seasonality. Are they enough to get a good picture of the influence of precipitation? In addition, the influence of rain-fed agriculture on WNFV indicates, that this could be important to look into.

- ***We agree with the reviewer that precipitation in spring and early summer could be useful for modelling WNF outbreaks. We included a comment regarding this matter in the first paragraph of Discussion (in the new clean version). However, these variables are not available in the CHELSA database. CHELSA supply data about precipitation in the wettest and driest months, which are different months in different parts of Europe. Nevertheless, we checked that these variables are correlated with annual precipitation and precipitation seasonality, which are more comparable between different European regions.***

Reviewer #2:

The manuscript “Predicting the spatio-temporal spread of the West Nile virus in Europe” is an original work aiming to provide insights on how environmental variables can be exploited to predict the spread of WNV in Europe. The topic is of interest. However, the length of the manuscript renders it quite difficult to focus and uncover the most important things. Additionally, several parts of the text are lengthy and irrelevant to the study – e.g. the first paragraph of the introduction, other phrases within that section, etc. The discussion is also rather extended (9 pages) and requires its re-reading several times in order to extract the important findings of this work. I suggest that the authors rearrange their manuscript by keeping only the important parts of it.

- ***We have eliminated the first paragraph of the introduction to focus the attention directly on the WNF, and reorganized other parts of this section according to the reviewer’s suggestions. We have shortened and rearranged the discussion to highlight the important findings of this work. The discussion section was divided in subsections to facilitate the reading of the work and was reduced to 7 pages following the reviewer’s suggestion.***

Line 4. “West Nile virus” (and elsewhere in the manuscript).

- ***We corrected the mistake throughout the manuscript.***

Line 5. The term “final hosts” applies for parasites, wherein a “final” host is required to complete the biological cycle. The proper term here is “dead-end” or “incidental”.

- ***We corrected the term and replaced “final host” with “dead-end hosts” here and elsewhere.***

Line 6. “Which affects the nervous system causing the West Nile Fever (WNF), leading to death”. a) The fact that the virus affects the nervous system (a condition called West Nile neuroinvasive disease, WNND) is not the same as fever. b) The way this has been written implies that WNV infection has always a lethal outcome. Please rephrase and use proper medical reports to present this sentence properly. This also applies for lines 31-32.

- ***Following the suggested comment, we have rephrased the sentences in line 6 and 31-32 (in the new clean version) to present them properly.***

Lines 6-8. “Since the circulation of the virus in Europe in the 1950s, human cases have just increased, with 2018 having the highest number of cases registered to date”. The expression “human cases have just increased” is misleading, as “just” should refer to the last 25 years. The first large-scale epidemics in humans occurred in Romania during 1996, with 393 encephalitis cases in humans, including 16 deaths. Lineage 2 was first isolated in 2004 and increased numbers of cases due to this lineage were observed from 2010 onwards. This also applies for lines 32-33.

- ***We have rewritten the sentences to avoid misunderstanding (current lines 6-8 and 32-33; in the new clean version).***

Lines 44-54. This paragraph is irrelevant to the present study. The introduction should briefly summarize the current situation as well as what is lacking from literature regarding the studied topic.

- ***We have eliminated the first paragraph of the introduction to focus the attention directly on the WNF, and reorganized other parts of this section according to the reviewer’s suggestions.***

Line 55. “Flavivirus” and “Flaviviridae” should be italicized to comply with ICTV nomenclature rules.

- ***We have italicized the words to comply with ICTV nomenclature rules (current line 43; in the new clean version).***

Lines 60-61. Reference to non-existent vaccines and drugs is not associated with the study.

- ***In this new version, we have rephrased the sentence to suggest that the lack of vaccines or drugs may render the study of the environmental correlates of the disease more important for prevention (current line 50; in the new clean version).***

Lines 55-64. I suggest to reorganize and simplify this paragraph, by mentioning firstly how the virus is being transmitted, secondly the hosts, and lastly the condition in infected humans.

- ***We have reorganized and simplified that paragraph following the reviewer’s suggestion (first paragraph in the Introduction; in the new clean version).***

Lines 65-77. This paragraph is also lengthy. The authors should help the reader focus to the main targets of their work and introduction should be written in a way to assist to this process. The fact, for example, that WNV took its name by the West Nile district of Uganda is distracting, not to mention that it is widely known.

- ***Given that our work provides a biogeographical perspective, those aspects related to the geographical and temporal distribution of the disease are critical to understanding our work. This is why we gave details about these distributions. Nevertheless, we have deleted the sentence regarding the origin of the name and we have shortened the paragraph to allow the reader to focus to the main targets of the work (second paragraph in the Introduction; in the new clean version).***

Line 119. “WNV-specific IgM”, etc. Use “serum (or virus) neutralization test” instead of “neutralization”.

- ***We have rewritten the sentence following the reviewer’s suggestion (current line 105; in the new clean version)***

Line 275. “Rain-fed agriculture, as well as poultry and horse livestock were the human activities that also contributed to explain the distribution of the WNF” How do the authors interpret the finding regarding horses? Other factors are directly associated with the vector or the propagation of the virus itself (birds). So, could there be an explanation or hypothesis for this finding?

- ***Regarding horses, we discussed in the Discussion section the possible role of horses (line 443; in the new clean version): “****Horse density also appears to favor the outbreak of the disease. This may be caused by the fact that equestrian farms provide suitable environments and breeding sites for the proliferation of mosquitoes, water troughs, ponds and purines being the most favorable habitats for the development of mosquito larvae* [2]*. C. pipiens is the principal vector of the WNV in Europe* [3] *and the most abundant mosquito species, by far, in equestrian farms* [2]***”***

Line 517. “Horse density also appears to favor the outbreak of the disease. This may be caused by the fact that equids are also dead-end hosts of the virus cycle.” How can this be supported? In horses the virus acts as in humans (dead-end) and mosquitoes cannot get infected by an infected horse. So, the dead-end character of the infection of horses (and humans), which is an accidental thing, is not expected to favor the outbreaks.

- ***We have corrected the sentence to connect horse density only with the proliferation of vector mosquitoes.***
