# Supplementary material for: Predicting the spatio-temporal spread of West Nile virus in Europe
Source: PLoS Negl Trop Dis. 2021 Jan 7;15(1):e0009022. doi: 10.1371/journal.pntd.0009022 (PMC7790247; doi:10.1371/journal.pntd.0009022)
Supplement: Attachment — Submitted filename: Authors response.docx [file pntd.0009022.s006.docx]

Dear Editors:

We are submitting our revised manuscript, in which we added additional explanations in response to the insightful comments and advices given by the reviewers. We highly appreciate their contributions because taken them into account we have improved the manuscript, both the scope and readability.

Below you will find the comments, followed by our response in italicized and bold type.

With kind regards, also on behalf of the authors,

Reviewer #3: Minor points:

- Delete the article in front of WNV and WNF throughout the entire document. Example: on line 4 replace "The West Nile virus ...." with "West Nile virus ....", on lines 46-47 replace "When infected by the WNV ...." with "When infected by WNV .... ", line 48 replace" that is known as the West Nile fever (WNF) "with" that is known as West Nile fever (WNF) ", or on line 312 replace" the WNF cases ..." by "WNF cases ... "

- ***Following the suggested comment, we have changed it throughout the document.***

- line 8: better replace “in Europe” with “the continent” in order not to repeat “Europe” so many times.

- ***We have rewritten the sentence, as suggested by the reviewer.***

- Replace throughout the document "the Julian week" by "the July week"

- ***When we refer to “Julian week” we do not mean the weeks of July, but the continuous count of weeks since the beginning of the year. That is why we keep “Julian week” throughout the manuscript.***

- line 33: “being 2018 the year with…”

- ***Done.***

- lines 60-62: (The transmission season of 2018 was exceptional; 1,605 cases were confirmed, which is twice as high as the sum of cases recorded in the previous three years): better “The transmission season of 2018 was exceptional; 1,605 cases were confirmed, which is double the sum of cases registered in the previous three years”

- ***We have rewritten the sentence.***

- line 72: “…are helpful for predicting the risk for WNF outbreaks” replace by “… are useful for predicting the risk of WNF outbreaks.”

- ***Following the suggested comment, we have replaced it.***

- line 154: (“…added at each step if and only if the resulting new regression was…”) delete “if and”

- ***Done.***

- line 243: a comma is needed (“…the higher the spatial and environmental favorability, the earlier the cases would…”)

- ***Done.***

- lines 270, 285: delete “Error! Reference source not found.”

- ***We have deleted it.***

- line 424: change “Other studies also showed that the WNF incidence had a…” by “Other studies also showed that the incidence of WNF had a…”.

- ***We have changed it.***
